# A static multi‐slit collimator system for scatter reduction in cone‐beam CT

**DOI:** 10.1120/jacmp.v11i4.3269

**Published:** 2010-10-07

**Authors:** Jina Chang, Siyong Kim, Doh‐Yun Jang, Tae‐Suk Suh

**Affiliations:** ^1^ Dept. of Biomedical Engineering College of Medicine, Catholic University Seoul 137‐701 Korea; ^2^ Dept. of Radiation Oncology Mayo Clinic Jacksonville FL 32224 USA; ^3^ Dept. of Nuclear Engineering Hanyang University Seoul 133‐791 Korea

**Keywords:** multiple‐slit, scatter reduction, cone‐beam CT

## Abstract

A multiple‐slit collimator (MSC) design was introduced for scatter reduction in cone‐beam computed tomography (CBCT). Unlike most other collimators, the open and closed septa of the proposed MSC are placed in an equi‐angular interval on a circular track of the central sagittal plane. Therefore, one gantry rotation provides only the half of necessary dataset and two gantry rotations are needed to obtain full information. During the first gantry rotation, the MSC position relative to the source is fixed. For the second rotation, the MSC is rotated by the equi‐angle interval. We assume signals under the closed septa are totally attributed to scatter radiation. Then, scatter contributions under open septa are determined by interpolating them.

Monte Carlo (MC) simulations for two virtual phantoms (one with a simple geometry and the other with two heterogeneities simulating the bone and the lung) were performed to evaluate the effectiveness of the system. Using the method developed, we could obtain images with significant scatter reduction. Contrast ratio (CR) improvement factors were 1.165 in a 2D projection view, and 1.210 and 1.223 at the central and peripheral slice of the reconstructed CBCT image of the simple geometry phantom.

This preliminary study demonstrated that the proposed MSC, together with the imaging process technique, had a great potential to reduce scatter contribution in CBCT. Further studies will be performed to investigate the effect of various factors, such as reducing the detector size, increasing the number of history of MC simulation, and including many structures with different densities.

PACS number: 87.57.C

## I. INTRODUCTION

Image‐guided radiation therapy (IGRT) is a state‐of‐the‐art treatment method that utilizes various imaging techniques to precisely deliver the intended dose to the treatment target. One of the most widely used imaging techniques for IGRT is cone‐beam CT (CBCT), in which a volumetric image set is reconstructed using many projected radiographic images under cone‐beam geometry.^(^ ^1^ ^–^ ^5^ ^)^

CBCT is a convenient and efficient imaging technique for radiation therapy treatments. However, the increased amount of scatter radiations due to the large projection field size of cone‐beam geometry reduces image contrast, increases image noise, and generates artifacts in reconstructed 3D images.^(^ ^6^ ^–^ ^7^ ^)^

Numerous strategies for scatter correction and rejection have been reported. It was demonstrated that compensation filters, so‐called bowtie filters, could reduce scatter and patient dose by compensating for the variation of attenuation through different patient body parts.^(^ ^8^ ^–^ ^9^ ^)^ In several scatter correction algorithms, scatter correction factors were obtained from pre‐acquired 2D scatter fluence maps and applied to each projection in CBCT to reduce scatter effect.^(^ ^10^ ^–^ ^12^ ^)^ A few studies showed the use of air gap and/or an antiscatter grid was very practical.^(^ ^13^ ^–^ ^15^ ^)^ By increasing the patient‐to‐detector distance and using a grid, a fair amount of scatter radiation was reduced. However, there were some limitations such as increased patient dose due to the attenuation of primary beam, focal spot blurring effect, and reduced field‐of‐view (FOV). A couple of slot‐scan methods employing single narrow fan beams^(^ ^16^ ^–^ ^18^ ^)^ were used for scatter reduction. Scatter radiations from neighboring beams were minimized by a dynamic fore‐ and after‐slit collimation system. Studies by Yester et al.^(^ ^19^ ^)^ and Barnes et al.^(^ ^20^ ^)^ introduced a multiple‐slit system in mammography to overcome the defect of single slit system. The multiple‐slit system could reduce the exposure. But its advantage was limited by the geometric dullness of the slit edges in mammography imaging.

One of the major advantages of slot method is the effectiveness of scatter reduction without the attenuation of primary X‐rays. However, the system requires the accurate alignment of the fore‐slit with the X‐ray focal spot, and the synchronization of the fore‐ and after‐collimator. To overcome these hardware difficulties, electronic collimation in a slit scan was proposed by Lie et al.^(^ ^21^ ^)^ They introduced electronic after‐collimation using the line erasure and readout (ALER) technique in a chest imaging. With this technique, the investigators were able to erase the scatter signals with no after‐collimator. However, even though this method eliminated hardware issues, it added another requirement – a specially designed ALER technique related to a flat‐panel digital radiography system.

In this study, we propose a static multiple‐slit system for the scatter reduction of CBCT. The proposed system consists of a fore‐multiple‐slit collimator and a scatter reduction imaging process. As a preliminary study, we performed Monte Carlo (MC) simulations to evaluate the effectiveness of the system.

## II. MATERIALS AND METHODS

### A. Multi‐slit collimator (MSC)

**Figure 1:**
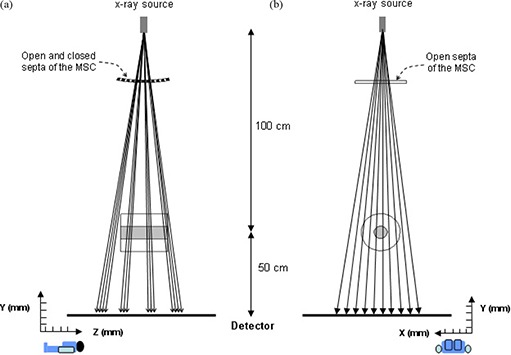
A simple diagram showing the multi‐slit collimator (MSC) and imaging geometry: (a) a sectional view on the central sagittal plane; (b) a sectional view on an axial plane corresponding to an open septum. As illustrated, the MSC consists of open and closed septa that are placed in equi‐angular interval on a circular track of the central sagittal plane.

Figure 1 shows a simple diagram of the MSC. As illustrated, open and closed septa are placed in equi‐angular interval on a circular track of the central sagittal plane. In this design, image acquisition is accomplished in two gantry rotations. In the first rotation, the MSC remains in a given position relative to the source and only half of the necessary dataset (corresponding to the object volume under the open septa) is obtained. Then the MSC is rotated on its circular track by the equi‐angle interval and another half of the dataset (corresponding to the object volume under the closed septa in the previous rotation) is acquired in the second gantry rotation.

### B. Monte Carlo simulation and imaging system

An MC software, MCNPX (v2.5d3, Los Alamos National Laboratory, Los Alamos, NM) program with the standard library of MCPLIB04 was used to build the imaging geometry and obtain 2D projection images. To evaluate the effect of scatter reduction in projection images, we used TIR radiography tally which was associated to flux image radiography on a planar image surface. The F5 TIR tally was also used to obtain both the scatter only radiation and primary plus scatter radiation. The number of particle histories was chosen large enough to be within 1% statistical noise of the MC calculation. The energy of X‐ray beam was 40 keV mono‐energetic. The distance between the source and the rotation center was 100 cm and the source‐to‐detector distance was 150 cm. Three slit widths, 10, 20, and 30 mm, were considered. The thickness of the slits, which were made of lead $\left(11.34g/{\text{cm}}^{3}\right)$, was 10 mm.

Two phantoms, one with a simple geometry (hereinafter referred to as ‘simple phantom’) and the other with heterogeneities simulating the lung and bone (hereinafter, ‘lung‐n‐bone phantom’), were considered. The simple phantom consisted of two regions, a PMMA $\left(1.19g/{\text{cm}}^{3}\right)$ outer cylinder of 15 cm diameter and a paraffin wax $\left(0.93g/{\text{cm}}^{3}\right)$ inner cylinder of 5 cm diameter. The length of the imaging object was 15 cm. The lung‐n‐bone phantom contained four regions: a PMMA $\left(1.19g/{\text{cm}}^{3}\right)$ outer cylinder of 15 cm diameter, two polyurethane ($\left(0.021g/{\text{cm}}^{3}\right)$) cylinders of 5 cm diameter to simulate both the right lung and the left lung, and a polytetrafluoroethylene (Teflon ‐ $\left(0.025g/{\text{cm}}^{3}\right)$) cylinder of 2 cm diameter to simulate the bone. The lengths of PMMA, polyurethane and Teflon regions were 20 cm, 9 cm, and 20 cm, respectively. The detector panel was $30\times 30{\text{cm}}^{2}$ wide and had 128×128 pixels, resulting in a pixel size of 2.34×2.34mm ^2^.

### C. Scatter reduction procedure

**Figure 2:**
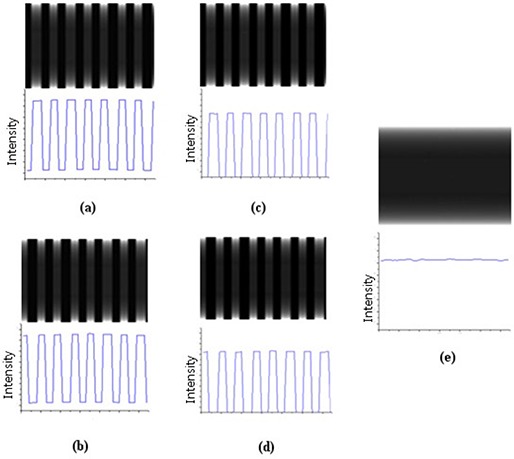
Scatter reduction scheme: (a) a raw projection image during the first gantry rotation; (b) a raw projection image during second rotation; (c) and (d) the projection images of the first and second rotation after subtraction of the scatter signals that are approximated by interpolating (and/or extrapolating) scatter signals under the closed septa; (e) the sum of images (c) and (d).

Figure 2 illustrates the scatter reduction scheme by showing how a final projection image corresponding to a given angle (i.e., gantry angle) is obtained. Figure 2(a) shows a raw projection image and its profile obtained during the first gantry rotation. Figure 2(b) shows a raw projection image and its profile obtained during second gantry rotation. As can be seen in each raw projection, signals are small under the closed septa but not zero and these are considered to be due to scattered radiation contribution. Next, we assume scatter signals in areas under the open septa can be approximated by interpolating (and/or extrapolating) scatter signals under the closed septa. These interpolated scatter signals are subtracted from the raw signals (see Figs. 2(c) and (d)). Finally, both scatter eliminated projection signals are added to get the final projection image (Fig. 2(e)).

### D. Evaluation of scatter reduction

To evaluate the effectiveness of scatter reduction, the scatter reduced projection images were compared with both the primary only and primary plus scatter radiation images. The primary radiation only image was obtained by subtracting the scatter only radiation values from primary plus scatter radiation values of the MC calculation.

We also estimated how much contrast ratio was improved. The image contrast ratio (CR) was defined as the ratio of intensity difference between a region and its surrounding region to the intensity of the surrounding region. With the simple phantom, for example, CR was calculated as follows:

(1) $$CR=\frac{\left|I^{P}‐I^{W}\right|}{I^{P}}$$

where, $I^{P}$ and $I^{W}$ were the intensity of the PMMA and the paraffin wax region, respectively.

In this study, the image reconstruction of CBCT was performed based on the Feldkamp, Davis, and Kress (FDK) reconstruction algorithm,^(^ ^22^ ^)^ with a total of 360 projection views.

## III. RESULTS

**Figure 3:**
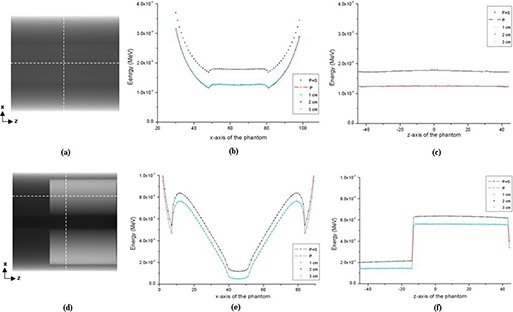
Anterior‐to‐posterior projection images and their profiles: (a) projection image of the simple phantom and its x‐axis profile (b) and its z‐axis profile (c); (d) projection image of the lung‐n‐bone phantom and its x‐axis profile (e) and its z‐axis profile (f). [‘P+S’, ‘P’, ‘1 cm’, ‘2 cm’, and ‘3 cm’ indicate primary plus scatter, primary only, 1 cm width MSC, 2 cm width MSC, and 3 cm width MSC, respectively.]

**Figure 4:**
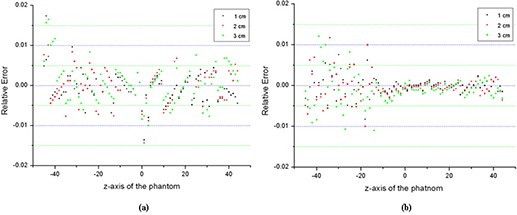
Relative errors of the obtained profiles in with respect to the primary radiation only profiles following the z‐axis: (a) the simple phantom, and (b) the lung‐n‐bone phantom. [‘1 cm’, ‘2 cm’, and ‘3 cm’ indicate 1 cm width MSC, 2 cm width MSC, and 3 cm width MSC, respectively.]

Figure 3 shows two anterior‐to‐posterior projection images, one for the simple phantom (Fig. 3(a) and the other for the lung‐n‐bone phantom (Fig. 3 (d)). Profiles in the x‐axis (lateral axis) and z‐axis (longitudinal axis) obtained following the white dotted lines are also shown. [Figs. 3(b) and (c) are for the simple phantom; Figs. 3(e) and (f) are for the lung‐n‐bone phantom.] As can be seen in Fig. 3, the MSC system effectively reduced scatter contributions for every size of the slit width considered, with the result that the profiles with the MSC were comparable to the primary only profile. In detail, Fig. 4 shows the relative errors of the obtained profiles in Fig. 3 with respect to the primary radiation only profiles following the z‐axis of the test phantoms. As shown in Fig. 4, the relative errors were within 2% for all slit widths in both phantoms. It is considered that the smaller the width, the better the effect of scatter reduction. However, it was observed that all sizes of the slit width provided similar result of scatter reduction in this study. This was mainly because: 1) the simulated phantom was relatively uniform, and 2) the distance between the phantom and the detector was far enough to make scatter contribution fairly flat over the detector.

**Figure 5:**
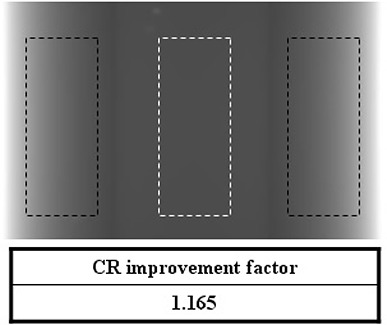
CR improvement factor of a 2D projection image for the simple phantom with the dotted regions representing the CR calculation regions (black: PMMA, white: paraffin).

Figure 5 shows the CR improvement factor of a 2D projection image for the simple phantom. The dotted regions represent the CR calculation regions (black: PMMA, white: paraffin). The CR improvement factor of the 2D projection view was 1.165.

**Figure 6:**
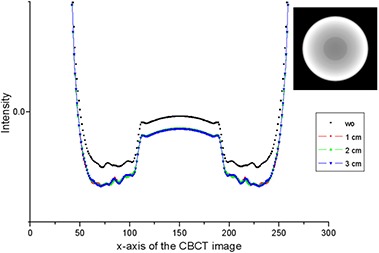
Central‐line intensity profile at the center slice of the reconstructed CBCT image of the simple phantom [‘wo’, ‘1 cm’, ‘2 cm’, and ‘3 cm’ indicate no MSC, 1 cm width MSC, 2 cm width MSC, and 3 cm width MSC, respectively.]

Figure 6 shows the central‐line intensity profile at the center slice of the reconstructed CBCT image of the simple phantom for each slit width. The case of no MSC (noted as ‘wo’) is also shown. Significant profile change can be observed in Fig. 6.

**Figure 7:**
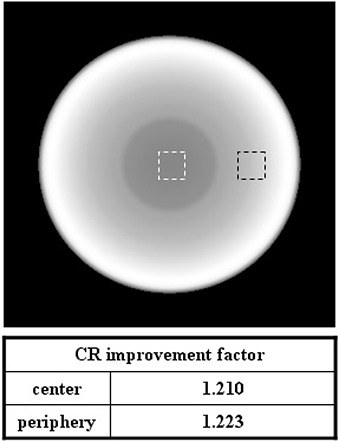
CR improvement factor of the CBCT image for the simple phantom; the two dotted boxes indicate the CR calculation regions.

Figure 7 shows the CR improvement factor of the CBCT image for the simple phantom. The CR improvement factors were 1.210 and 1.223 at the central and peripheral slice of the CBCT image set, respectively. Two dotted boxes indicate the CR calculation regions.

**Figure 8:**
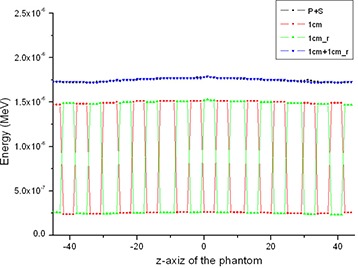
Energy profile (in z‐direction) that the detector received in the case of 1 cm width MSC: [‘P+S’ indicates primary plus scatter, ‘1 cm’ and ‘1cm_r’ indicate two projections of a pair obtained in a given angle (i.e. one in the first MSC position and the other with the MSC rotated by one equi‐angle interval), and ‘1cm+1cm_r’ represents the summation of them without any image processing.]

To indirectly evaluate detector's radiation damage, we compared the amount of energy that the detector received with and without the MSC. Figure 8 shows the energy profile (in z‐direction) the detector received in the case of 1 cm slit width. The labels, ‘1 cm’ and ‘1cm_r’ indicate two projections of a pair obtained in a given angle (i.e. one in the first MSC position and the other with the MSC rotated by one equi‐angle interval). And ‘1cm+1cm_r’ represents the summation of them without any image processing. The result shows that the amount of energy the detector received with the MSC was comparable to that with the conventional projection. This result indicates dose increase caused by the MSC system and its application is negligible.

## IV. DISCUSSION

We have proposed a new design of multi‐slit collimator and a simple method of scatter reduction to accompany it. The virtual evaluation of the system was performed using MCNP simulations with radiography tally 5 that enabled both scatter only and primary plus scatter calculations. The simulation results demonstrated that the system could provide significant scatter reduction.

Typical dynamic slit systems require the accurate alignment of fore‐ and after‐collimator during image acquisition. However, the design of our system requires the simple hardware configuration of static multiple fore‐collimator only, and can be easily adapted to conventional x‐ray systems. In addition, the static detector system is convenient for image processing.

There are a few weaknesses of the system. First, there could be penumbra effect between two projection images due to imperfect matching in real environment. However, it could be minimized with both elaborate engineering and image processing. Second, increased scan time can potentially degrade image quality due to possible increased patient motion. Although we believe these issues can be minimized with enhanced immobilization techniques, they should be investigated and will be subjects for future study. The final problem is possible increased patient dose due to the scattered radiation of the MSC and the leakage radiation of the second gantry rotation. However, we believe the amount of dose increase would be very small because typical leakage is less than 0.1% and most photons hitting the collimator are absorbed. In addition, the significant scatter reduction of the MSC system could enhance the CBCT image quality, and this could make it possible to reduce the number of projections to reconstruct CT image without image quality degradation. By reducing the number of projections in CBCT, it may be possible to lower the patient dose.

Considering that typical kVp value in CBCT ranges from 100 kVp to 125 kVp, we chose 40 keV mono‐energy photons in this study. Scatter radiation is produced mainly when Compton scattering interactions occur and the Compton scattering cross section of water (or tissue) is higher with higher photon energy in the range of CBCT x‐ray. Because we are considering scatter reduction effect, it would be more conservative to take the lower side of photon energy. We believe 40 keV is rather lower than actual and a conservative choice. Further study will be performed to simulate a more realistic CBCT x‐ray system including the target and filter materials, and investigate the effect of various factors such as reducing the detector size, increasing the number of history of MC simulation, and including various structures with different densities.

## V. CONCLUSIONS

We have evaluated the scatter reduction effect of the multi‐slit collimator system in CBCT imaging using the MC simulations. Preliminary study based on the MCNP simulations showed a significant scatter reduction could be achieved with the proposed system.

## ACKNOWLEDGEMENTS

This research was sponsored by the National Research Foundation of the Ministry of Education, Science and Technology of Korea (2010‐0003315, K20901000001‐09E0100‐00110).
